# Supplementary material for: Substrate composition directs slime molds behavior
Source: Sci Rep. 2019 Oct 28;9:15444. doi: 10.1038/s41598-019-50872-z (PMC6817824; doi:10.1038/s41598-019-50872-z)
Supplement: Supplementary file 1 — Supplementary Information [file 41598_2019_50872_MOESM1_ESM.pdf]

Morphogenesis and dynamics of slime molds in various environments

Supplementary information

Fernando Patino-Ramirez [1*], Aurèle Boussard[2], Chloé Arson[1¶], Audrey Dussutour[2¶*]

[1] School of Civil and Environmental Engineering, Georgia Institute of Technology, Atlanta, GA, USA

[2] Research Centre on Animal Cognition (CRCA), Centre for Integrative Biology (CBI), Toulouse University, CNRS, UPS, Toulouse, France

* Corresponding authors

E-mails: fp@gatech.edu (FP), audrey.dussutour@univ-tlse3.fr (AD)

¶ These authors contributed equally to this work.

S1 Appendix: Supplementary information: image analysis and statistical results.

Contents

I. Computational methods: from image analysis to statistical procedure 2

1. Ratio of secondary and primary growth and ratio of neighboring mucus and unexplored pixels 4
2. Slime molds shape analysis 5
3. Analysis of temporal series: how we summarized temporal dynamics to answer statistical questions. 6

II. Homogeneous experiments: Model comparisons and effect size graphics 8

1. Slime mold: 8
   a) Time elapsed until the first movement 8
   b) Growth rate during the linear growth at the beginning 9
   c) Final surface 10
2. Mucus: 11
   a) Time elapsed until the first appearance 11
   b) Growth rate during the linear growth at the beginning 12
   c) Final surface 13
3. Total secondary growth 14
4. Total migration rate 15
5. Tendency for secondary growth 16
6. Solidity decrease rate 17
7. New pseudopod number 18

III. Spot experiments: Model comparisons and effect size graphics 19

1. Slime mold: 19
   a) Time elapsed until the first movement 19
   b) Growth rate during the linear growth at the beginning 21
   c) Final surface 21

2. Mucus: 22
a) Time elapsed until the first appearance 22
b) Growth rate during the linear growth at the beginning 23
c) Final surface 24
3. Total secondary growth 25
4. Total migration rate 25
5. Tendency for secondary growth 27
6. Solidity decrease rate 28
7. New pseudopod number 29
8. Time to reach the food patch 29
IV. References: 31

I. Computational methods: from image analysis to statistical procedure

1. Image classification and trinarization.

In order to show the capabilities of the image analysis code developed in this study, and in order to give some insights to the reader about the behavior of the slime molds in the petri dish. Files S2_video and S3_video show the spatial evolution of the cell over time (35 hours), showing the original images and the identification results respectively.

2. Ratio of secondary and primary growth and ratio of neighboring mucus and unexplored pixels

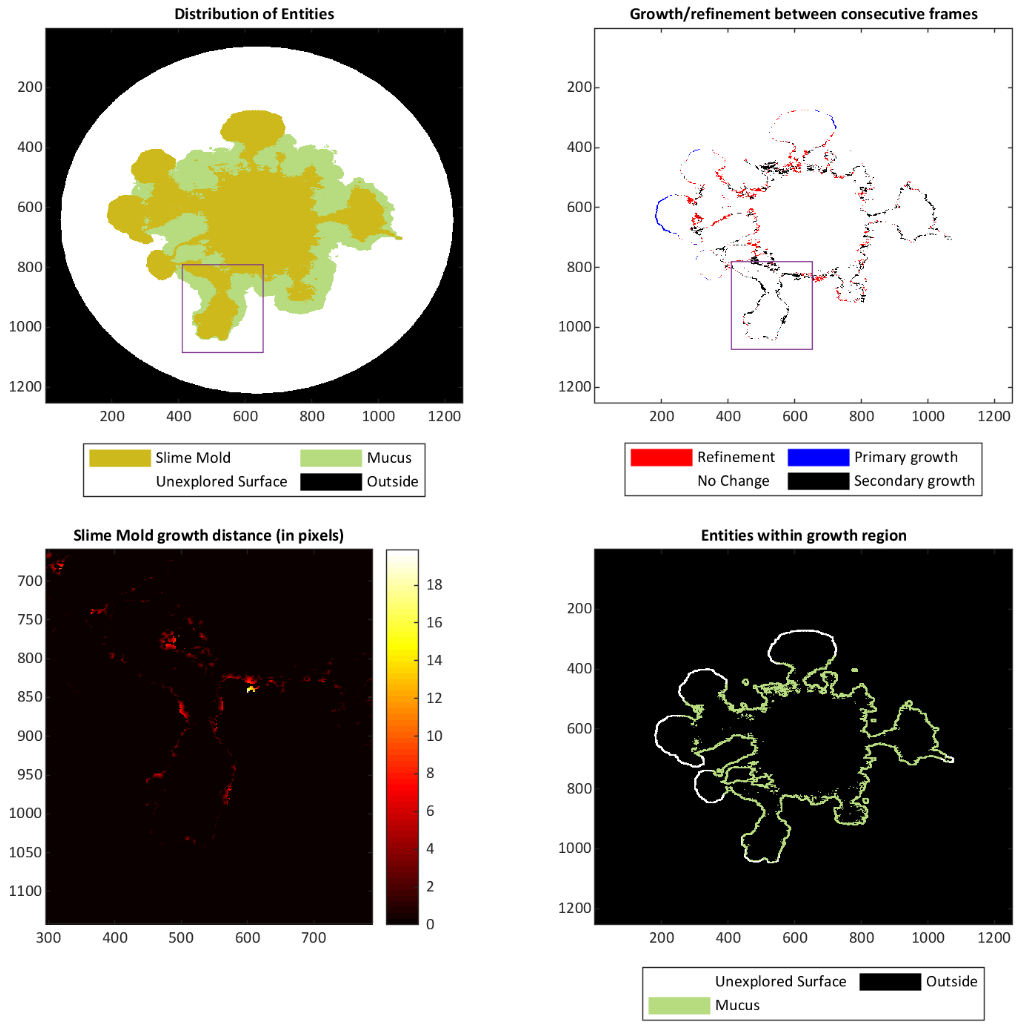

Figure 1: Process to find secondary growth ratio and mucus ratio. Top left: Sample trinarized image with 3 entities. Top Right: location Growth/refining pixels. Bottom left:

Distance from slime mold contour to growth pixels [px], the migration rate is defined as the maximum value. Bottom right: Neighboring area, built as an offset of width equal to the migration rate from the slime mold cell contour.

3. Slime molds shape analysis

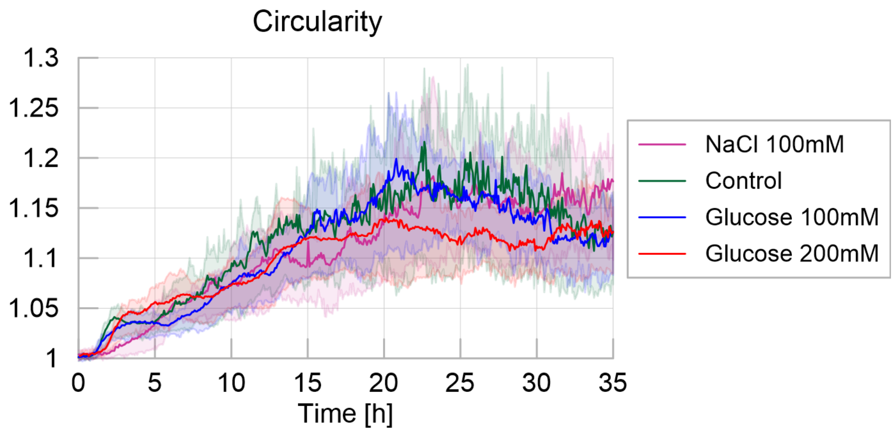

Figure 2. Circularity over time, homogeneous analysis: mean values (solid lines) and area enclosed by the first and third quartiles (shaded area) over 20 replicates per treatment. A value of 1 corresponds to a circle and diverges as it deviates from it.

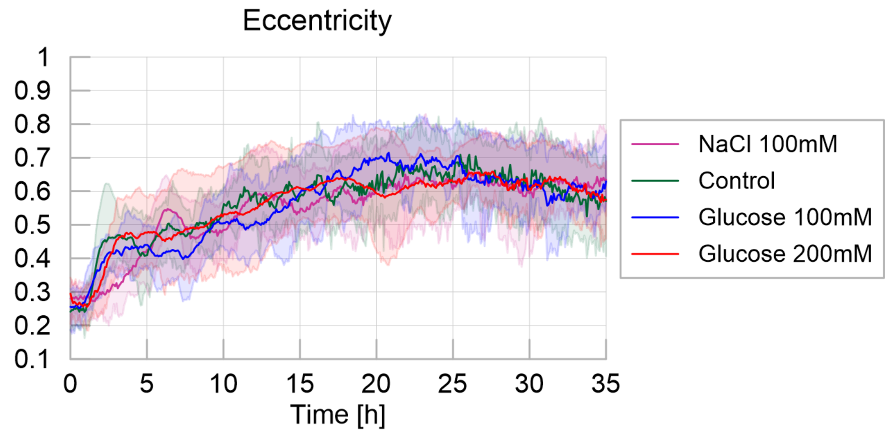

Figure 3. Eccentricity over time, homogeneous analysis: mean values (solid lines) and area enclosed by the first and third quartiles (shaded area) over 20 replicates per treatment. A value of 0 corresponds to a perfect circle and a value of 1 corresponds to a line (infinitely eccentric shape).

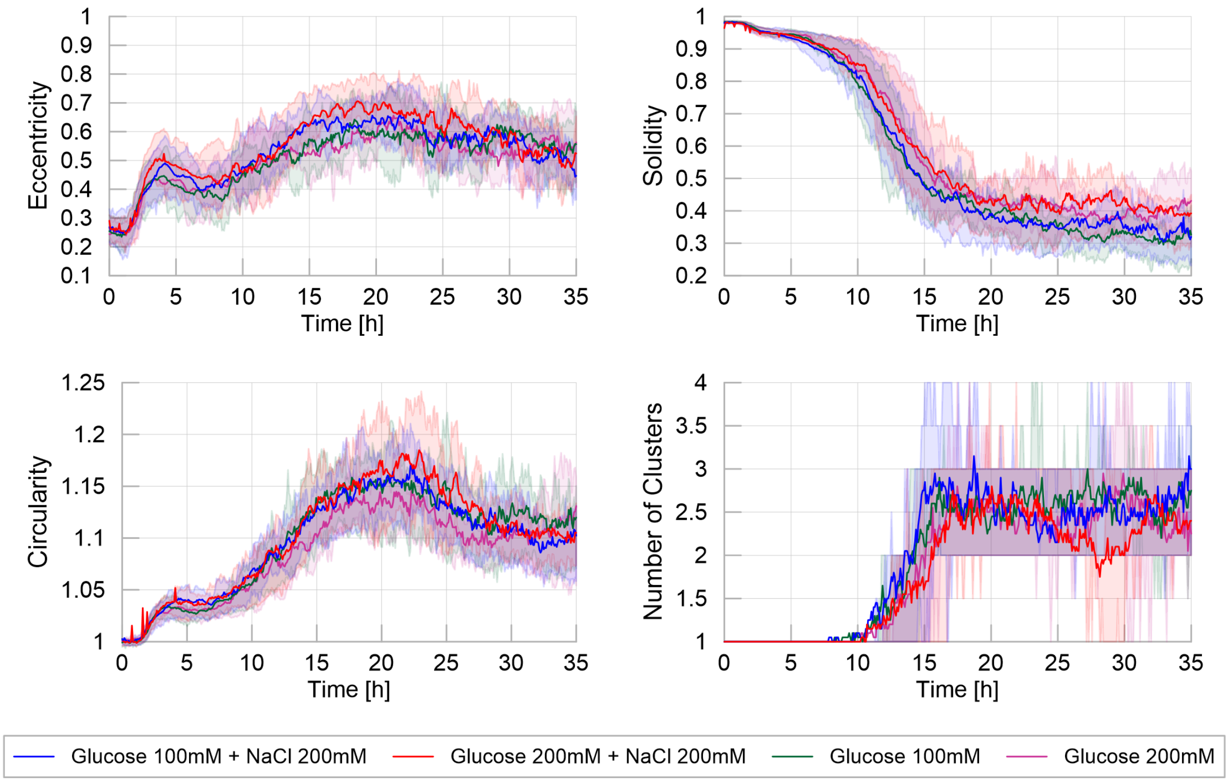

Figure 4: Shape indexes over time, spot analysis. Different treatments are shown as solid lines and shaded areas of similar color correspond to the first and third quartiles of the data (20 replicates per treatment)

4. Analysis of temporal series: how we summarized temporal dynamics to answer statistical questions.

We conducted a statistical analysis on the metrics that were extracted from image analysis to describe temporal dynamics. Since the eccentricity and circularity against time presented no clear pattern, we focused our analysis on the following dynamics: slime mold area (and more particularly, the cumulated primary and secondary growth), mucus area, slime mold contour solidity and distance of slime mold from the food patch. We chose to present descriptors that are easy to compare by using linear or survival analysis. For each growth dynamics, we used the programing language R [1] to compute the following dependent variables:

- Time elapsed before the first movement: we first calculated the difference between the largest and the smallest area of the entity studied for each replicate. We calculated the average value of that difference over the total number of

replicates. The first movement was defined as the first change of coverage area greater or equal to one percent of that average value. The time of occurrence of the first movement was determined from the number of the frame in which the first movement was detected.

- Time elapsed to reach a plateau: we calculated the average cumulated surface every 25 minutes for each replicate. Growth rate reduction was detected by calculating the difference between two consecutive mean cumulated surfaces. Finally, we got the time to reach a plateau when both such a slope reduction happened and when the surface was at least of 90 percent of the maximal variation. We interpreted curves that did not met these conditions as censored (i.e. when experiment duration was not long enough to detect this event) in our survival analysis.
- Linear growth rate: For each replicate, we assessed which possible time intervals would contain a growth increase larger than 50% of the total surface increase. Then, we computed linear regressions on these intervals and for each replicate, we extracted the growth rate from the regression having the highest r squared.
- Total surface: we took the last value of the cumulated surface for each individual (slime mold, mucus and unexplored substrate, for each replicate)
- Solidity decrease rate: we calculated this slope following the same method as for the growth rate of cumulated surfaces.
- New pseudopod number: for each replicate, we summed the total number of new pseudopods appearing every 5 minutes.
- Total tendency for secondary growth: for each time frame, we calculated the difference between the proportion of pixels covered by secondary growth and the proportion of neighboring mucus pixels. By summing these differences for each replicate, we got the slime mold tendency to do secondary growth instead of primary growth.
- To get the time to reach a food patch, we looked for the first time frame when the slime mold reached the food patch.

For the dependent variables defined as the time elapsed until an event happens, we did survival analyses using the R packages coxme [2] and survival [3]. For the other dependent variables, we did linear analyses using the R packages lme4 [4] and lmerTest [5]. In the homogeneous experiment (N=80) we tested the four different treatments (CONT when growth happened on a neutral substrate, G100, G200 and N100 when on glucose 100 mM, 200 mM or NaCl 100 mM respectively, n = 20 for each four treatments) as fixed factors. In the spot experiment (N=80) we tested the effect of the nature of the nearest spot (neutral (CONT) or NaCl 100mM (N100)), of the furthest spot (glucose 100mM (G100) or 200mM (G200), n = 20 for each four treatments) and interaction between these factors. Except for the study of the decrease rate of solidity in the homogeneous experiment, we always took the date as a random factor. For each statistical question, we performed nested model comparisons using the R package

MuMIn [6] by ordering models according to their Akaike criterion. We represented the selected models by plotting estimators with the pairwise comparison (a posteriori Tuckey test) p-values using the R package emmeans [7] in linear models and the hazard ratio associated p-values in cox models. In linear models, we marked each significant pairwise comparison by the p-value positioned in between the two compared dots and we interpreted a significant difference when a pairwise test revealed a p-value inferior to 5%. In cox models, we interpreted a significant difference from controls when a non-control treatment revealed a p-value inferior to 5%. We represented the degree of significance above each p-value with one star (*) if the p-value was lower than 5%, two stars (**) if lower than 1% and three stars (***) if lower than 0.1%.

II. Homogeneous experiments: Model comparisons and effect size graphics

1. Slime mold:

a) Time elapsed until the first movement

The surface threshold used to detect the slime mold first movement was 0.29 (pixels)

The cox model explaining the time for a first movement by the nature of the treatment respected the proportional hazard ratio assumption.

Table 1: Model selection (using AIC) of cox models explaining the time for a first movement by the nature of the treatment in the homogeneous experiments.

Treatment	R^2	df	logLik	AIC	delta
+	0.65	5	-232	472	0
0	0.01	0	-273	547	75

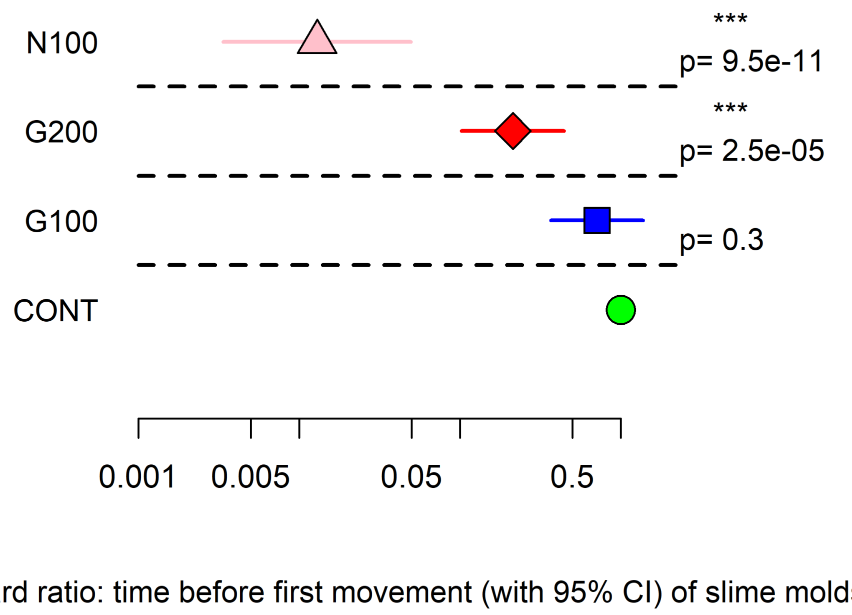

Figure 5: Hazard ratio (and 95% CI) of the fixed effects of the selected model. The lower the hazard ratio, the lower the risk –at any time- that the slime mold starts to move, i.e. the lower the hazard ratio, the more time needed for slime mold to move. When the substrate contains glucose 100mM, the time to start to move is not different from that in the control case, while it is higher when the substrate contains glucose 200mM and even higher when the substrate contains NaCl 200mM

b) Growth rate during the linear growth at the beginning

The linear mixed model explaining the growth rate by the four different treatments showed a slight deviation from homoscedasticity but linearity, independence, normality and absence of outliers was respected.

Table 2: Model selection (using AIC) of linear mixed models explaining the growth rate by the nature of the treatment in the homogeneous experiments.

Treatment	R^2 for fixed effects	R^2 for all effects	df	logLik	AIC	delta
+	0.26	0.34	6	244.3	-476.6	0.0
0	0.00	0.06	3	231.2	-456.3	20.3

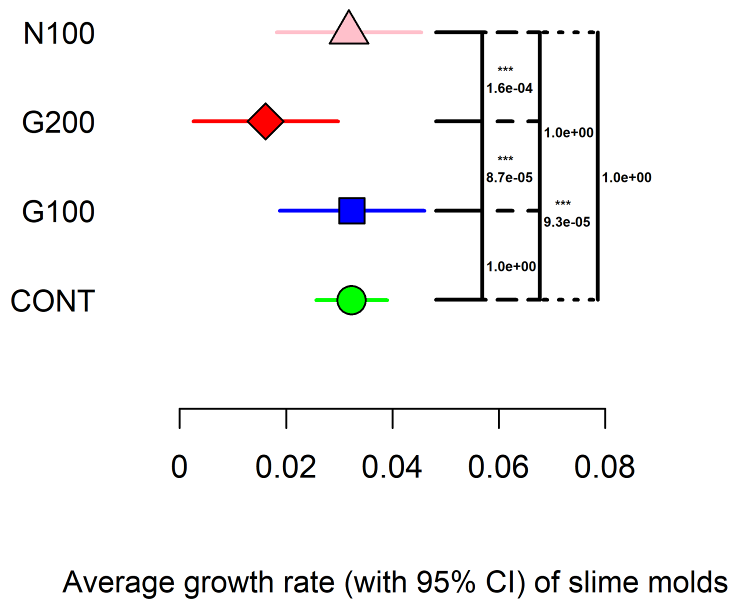

Figure 6: Average growth rate (and 95% CI) of the selected model. The lower the growth rate, the slower the exploration of the substrate. The dendrograms represent pairwise comparisons using a Tukey test. When the substrate contains glucose 100mM or NaCl 100mM, the explored surface does not increase slower than in the control case. However, when the substrate contains glucose 200mM, slime mold exploration is slower than any other treatments.

c) Final surface

The linear mixed model explaining the final surface by the four different treatments showed a slight deviation from linearity but homoscedasticity, independence, normality and absence of outliers was respected.

Table 3: Model selection (using AIC) of linear mixed models explaining the final surface of slime mold by the nature of the treatment in the homogeneous experiments

Treatment	R^2 for fixed effects	R^2 for all effects	df	logLik	AIC	delta
+	0.21	0.32	6	-287.1	586.2	0.0
0	0.00	0.11	3	-297.6	601.3	15.1

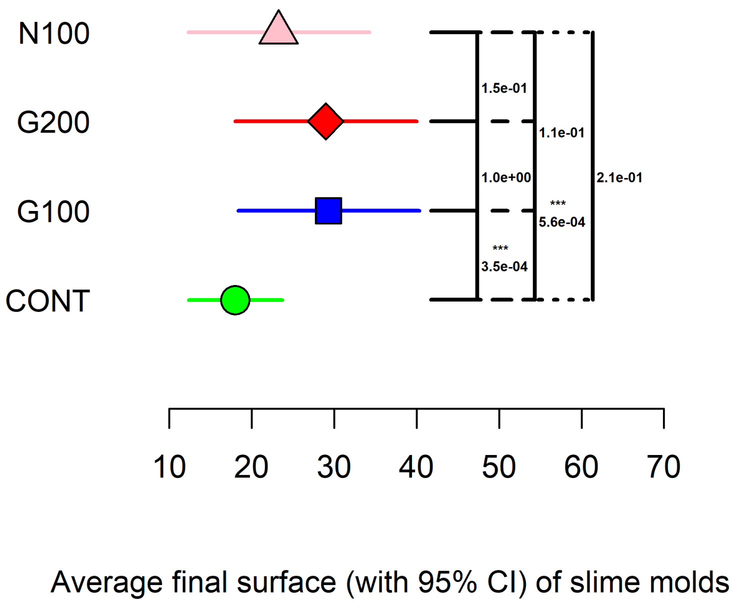

Figure 7: Average final surface (and 95% CI) of the selected model. The dendrograms represent pairwise comparisons using a Tukey test. The final surface of slime molds on both nutritive substrates is higher than on a neutral substrate. However, on an adverse substrate the final surface is not significantly different from the control case.

2. Mucus:

   a) Time elapsed until the first appearance

The surface threshold used to detect the first mucus production was 0.41 (pixels).

The cox model explaining the time elapsed until a first movement by the four different treatments respected the proportional hazard ratio assumption.

Table 4: Model selection (using AIC) of cox models explaining the time elapsed until the first mucus production by the nature of the treatment in the homogeneous experiments.

Treatment	R^2	df	logLik	AIC	delta
+	0.62	5	-235	479	0
0	0.33	2	-258	518	38

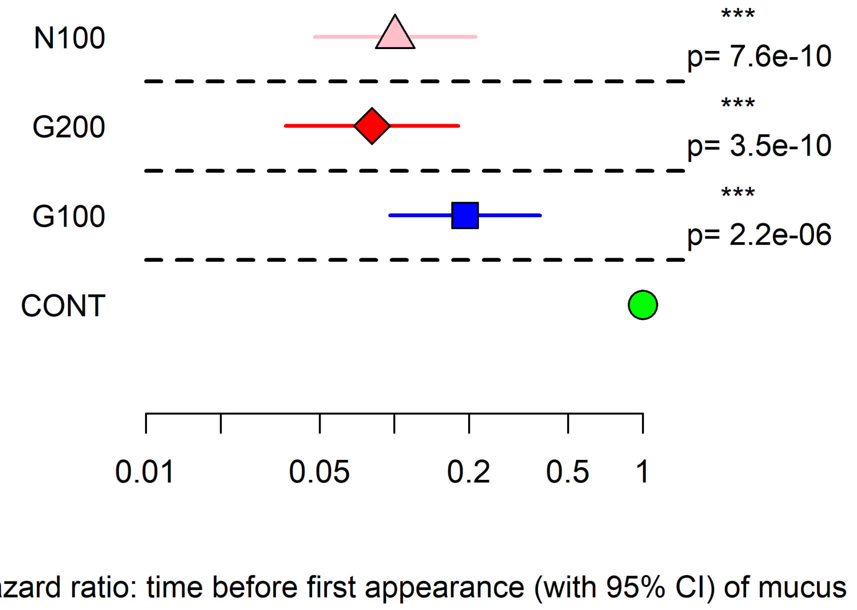

Figure 8: Hazard ratio (and 95% CI) of the fixed effects of the selected model. The lower the hazard ratio, the lower the risk –at any time- that mucus production starts, i.e. the lower the hazard ratio, the larger delay in mucus production. Compared to other treatments, mucus production takes less time to start when the control substrate contains agar only. Glucose and sodium chloride delay mucus production.

While a substrate containing 100mM glucose did not delay slime mold growth, it did delay mucus production. On the other hand, 200mM glucose and 100 mM NaCl delayed both slime mold growth and mucus production.

b) Growth rate during the linear growth at the beginning

The linear mixed model explaining the growth rate by the four different treatments respected linear mixed model assumptions.

Table 5: Model selection (using AIC) of linear mixed models explaining the linear growth rate variations by the nature of the treatment in the homogeneous experiments

Treatment	R^2 for fixed effects	R^2 for all effects	df	logLik	AIC	delta
+	0.66	0.69	6	217.1	-422.3	0
0	0.00	0.00	3	173.1	-340.2	82

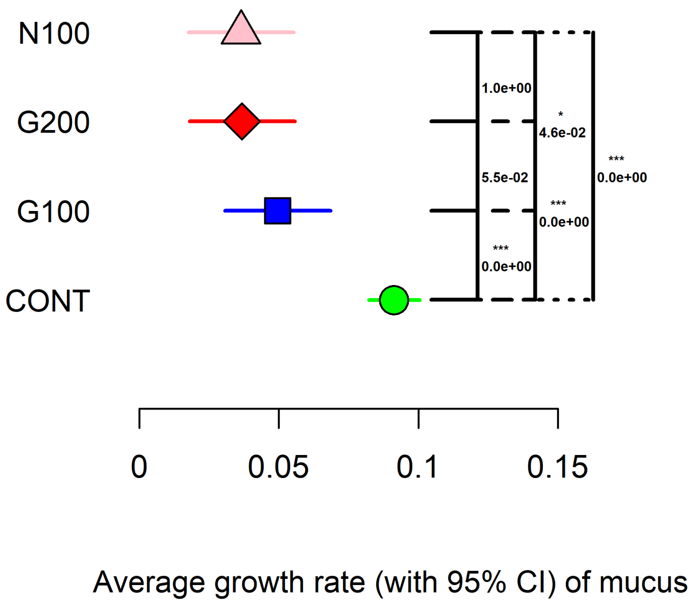

Figure 9: Average growth rate (and 95% CI) of the selected model. The lower is the growth rate, the slower is the mucus covering of the substrate. The dendrograms represent pairwise comparisons using a Tukey test. Mucus covering is faster on the neutral substrate than on any other treatment and it is slower on an adverse substrate than on a slightly nutritive one.

c) Final surface

The model explaining the final surface by the four different treatments respected the linear mixed model assumptions.

Table 6: Model selection (using AIC) of linear mixed models explaining the final surface of mucus by the nature of treatment in the homogeneous experiments.

Treatment	R^2 for fixed effects	R^2 for all effects	df	logLik	AIC	delta
+	0.6	0.62	6	-313.2	638.4	0.0
0	0.0	0.00	3	-349.8	705.6	67.3

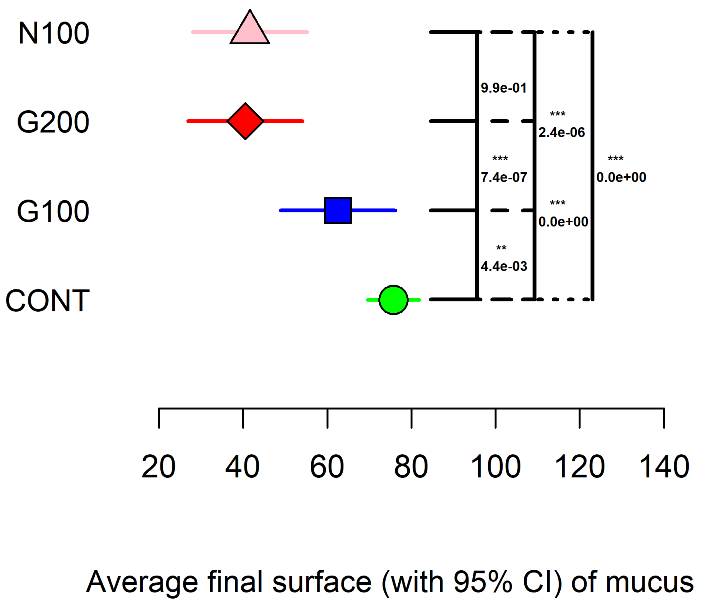

Figure 10: Average final surface (and 95% CI) of the selected model. The dendrograms represent pairwise comparisons using a Tukey test. The final surface of mucus is higher on a neutral substrate than on every other substrate. The final mucus surface is the same in the adverse environment and in the highly nutritive environment. Furthermore, on both of these substrates, the final mucus surface is lower than in the slightly nutritive substrate.

3. Total secondary growth

Concerning the linear mixed model explaining the total secondary growth by the four different treatments, a log transformation allowed to correct a deviation from homoscedasticity.

Table 7: Model selection (using AIC) of linear mixed models explaining the total secondary growth by the nature of the treatment in the homogeneous experiments.

Treatment	R^2 for fixed effects	R^2 for all effects	df	logLik	AIC	delta
+	0.42	0.50	6	-36.7	85.5	0.0
0	0.00	0.06	3	-60.7	127.3	41.8

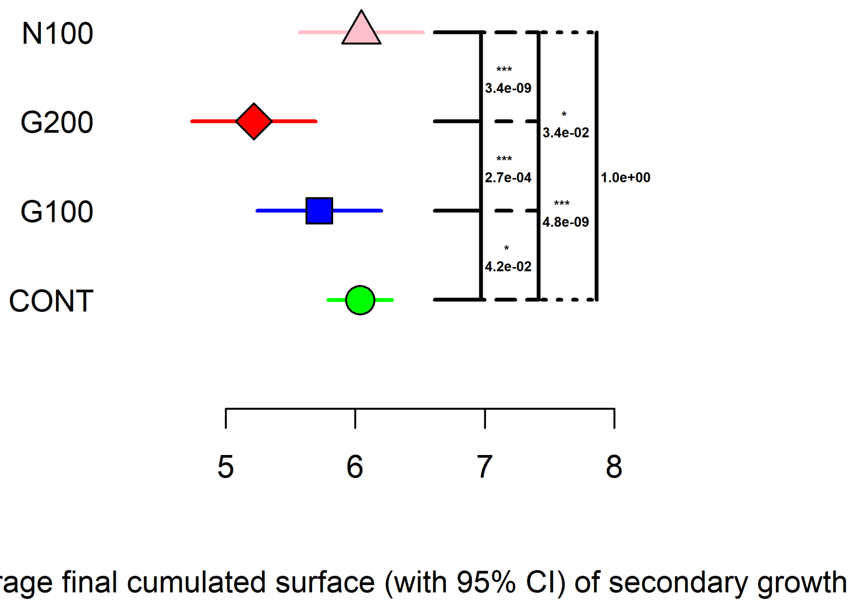

Figure 11: Average total secondary growth (and 95% CI) of the selected model. The higher this cumulated surface, the higher the number of times slime molds visited an already explored surface. The dendrograms represent pairwise comparisons using a Tukey test. This cumulated surface is the highest on the neutral substrate and on the substrate containing salt. It is lower when the substrate contains 100mM glucose and even lower when it contains 200 mM glucose.

4. Total migration rate

The linear mixed model explaining the total migration rate showed no deviation from linear mixed models assumptions.

Table 8: Model selection (using AIC) of linear mixed models explaining the total migration rate by the nature of the treatment in the homogeneous experiments.

Treatment	R^2 for fixed effects	R^2 for all effects	df	logLik	AIC	delta
+	0.58	0.71	6	-394.5	801.0	0.0
0	0.00	0.11	3	-437.3	880.6	79.5

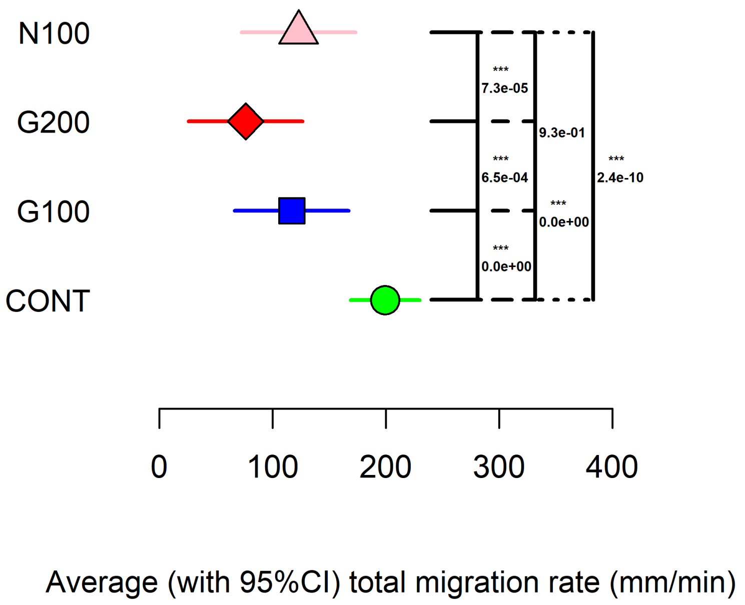

Figure 12: Average total migration rate (and 95% CI) of the selected model. The higher the total migration rate, the more slime molds explored their environment. The dendrograms represent pairwise comparisons using a Tukey test. Total migration rate was the highest on a neutral environment and the lowest on a highly nutritive environment. There were no significant differences between an adverse and a slightly nutritive environment.

5. Tendency for secondary growth

The linear mixed model explaining the tendency for secondary showed no deviation from linear mixed models assumptions.

Table 9: Model selection (using AIC) of linear mixed models explaining the tendency for secondary growth by the nature of the treatment in the homogeneous experiments.

Treatment	R^2 for fixed effects	R^2 for all effects	df	logLik	AIC	delta
+	0.63	0.71	6	-336	684	0
0	0.00	0.04	3	-381	768	84

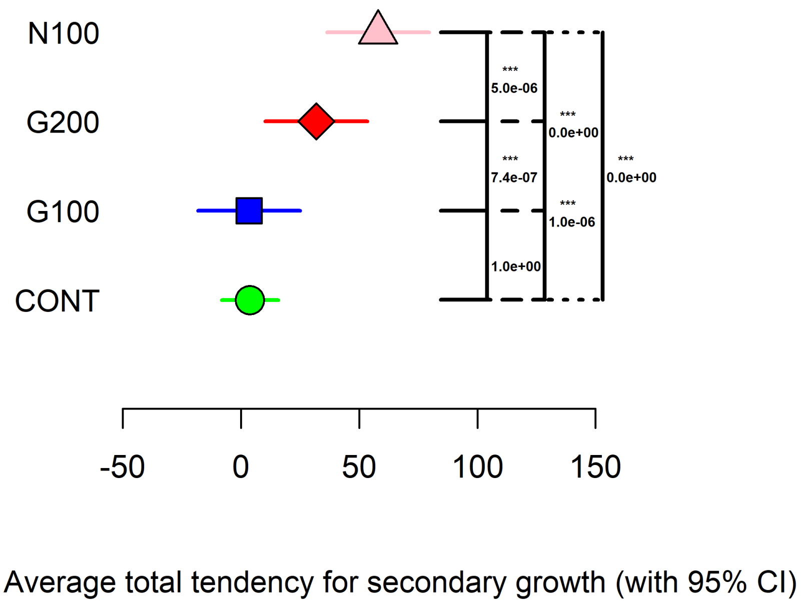

Figure 13: Average total tendency for secondary growth (and 95% CI) of the selected model. The higher the total migration rate, the more slime molds explored their environment. The dendrograms represent pairwise comparisons using a Tukey test. While the total tendency for secondary growth in a neutral and a slightly nutritive environment was null, it was significantly higher in a highly nutritive environment. The tendency for secondary growth was significantly higher in an adverse than in any other environment.

6. Solidity decrease rate

When studying the decrease rate of solidity, taking into account the date as a random factor produced singular fit. Consequently, we analyzed this rate using a linear model – the assumptions of which were respected.

Table 10: Model selection (using AIC) of linear models explaining variations in the decrease rate of solidity by the nature of the treatment in the homogeneous experiments.

Treatment	R^2	df	logLik	AIC	delta
+	0.57	5	527	-1043	0

0	0.00	2	493	-982	61

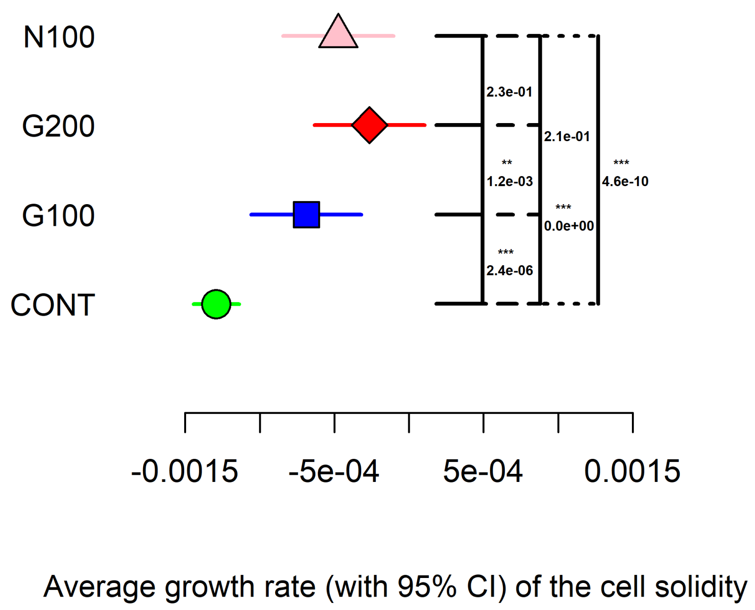

Figure 14: Average decrease rate (and 95% CI) of the selected model. The dendrograms represent pairwise comparisons using a Tukey test. The stronger decrease of rate happens on neutral substrates (agar). This rate is closer to 0 when the substrate contains 100mM glucose and even closer when it contains 100mM glucose (CI overlap 0) or 100mM NaCl.

7. New pseudopod number

When studying the new pseudopod number, the linear mixed model assumptions were respected.

Table 11: Model selection (using AIC) of linear mixed models explaining variations in the total of new pseudopods having appeared during the homogeneous experiment by the nature of the treatment.

Treatment	R^2 for fixed effects	R^2 for all effects	df	logLik	AIC	delta
+	0.41	0.50	6	-352.4	716.8	0.0
0	0.00	0.06	3	-376.0	758.1	41.3

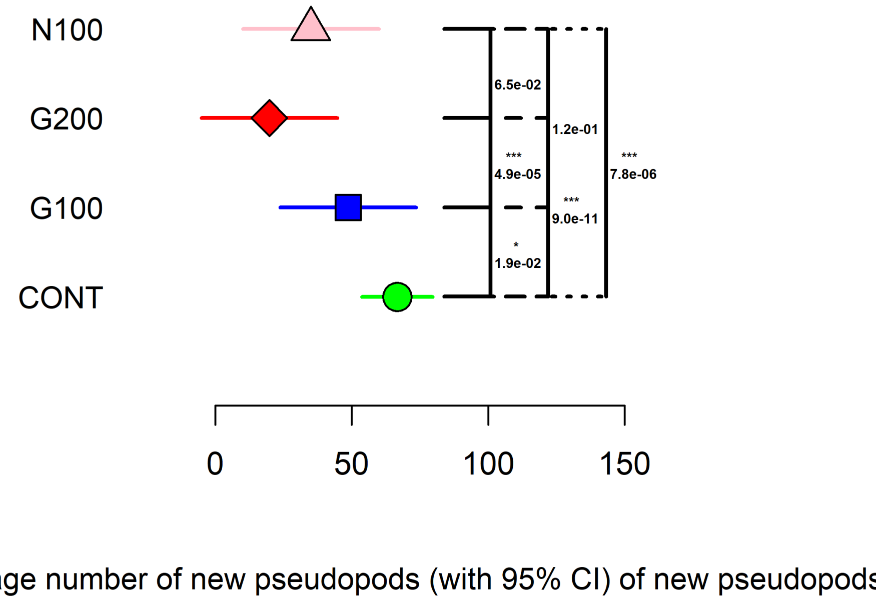

Figure 15: Average number of new pseudopods (and 95% CI) extracted from the selected model. The dendrograms represent pairwise comparisons using a Tukey test. The highest number of new pseudopods is observed in neutral environments. This number is lower in the slightly nutritive and in the adverse environments (which are not different) and even lower on the highly nutritive environment. However, there is no significant difference between the highly nutritive and the adverse environments

III. Spot experiments: Model comparisons and effect size graphics

1. Slime mold:
   a) Time elapsed until the first movement

The surface threshold used to detect the slime mold first movement was 0.21 (pixels)

The cox model explaining the time for a first movement by the treatments has a slight deviation from the proportional hazard ratio assumption.

Table 12: Model selection (using AIC) of cox models explaining the time elapsed until the first movement by the nature of the treatment in the spot experiments.

Food spot concentration	Obstacle	Food : Obstacle	R^2	df	logLik	AIC	delta
0	+	0	0.57	3	-240	484	0.0
+	+	0	0.57	4	-240	486	1.9
0	0	0	0.55	2	-242	486	2.2
+	+	+	0.58	5	-239	487	2.9
+	0	0	0.55	3	-242	488	4.2

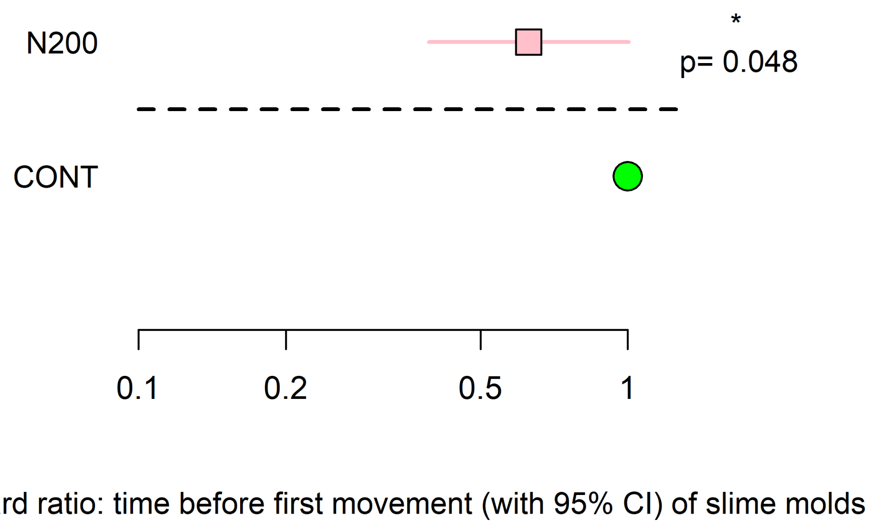

Figure 16: Hazard ratio (and 95% CI) of the fixed effects of the selected model. The lower the hazard ratio, the lower the risk –at any time- that the slime mold starts to move, i.e. the lower the hazard ratio, the more time needed for slime molds to move. When the substrate contains 100mM NaCl, the time to initiate movement is slightly lower than in control substrate. Other treatments are not different from the control.

Given the overlapping between the two confidence intervals, this result might be a false positive.

b) Growth rate during the linear growth at the beginning

The linear mixed model explaining the growth showed no deviation from linear mixed models assumptions.

Table 13: Model selection (using AIC) of linear mixed models explaining the growth rate by the nature of the treatment in the spot experiments.

Food spot concentration	Obstacle	Food : Obstacle	R^2 for fixed effects	R^2 for all effects	df	logLik	AIC	delta
0	+	0	0.01	0.63	4	264.6	-521.2	0.000
0	0	0	0.00	0.62	3	263.5	-521.0	0.164
+	+	+	0.02	0.64	6	266.0	-519.9	1.241
+	+	0	0.01	0.63	5	264.9	-519.9	1.305
+	0	0	0.00	0.62	4	263.8	-519.7	1.488

The AIC difference between the null model and the best model is very small. Consequently, the linear growth rate is not significantly impacted by the nature of the two spots.

c) Final surface

The linear mixed model explaining the final surface by the different treatments showed a slight deviation caused by an extreme value but linearity homoscedasticity, independence and normality were respected.

Table 14: Model selection (using AIC) of linear mixed models explaining the final surface of slime mold by the nature of the treatment in spot experiments

Food spot concentration	Obstacle	Food : Obstacle	R^2 for fixed effects	R^2 for all effects	df	logLik	AIC	delta
+	0	0	0.12	0.16	4	-291.0	589.9	0.0
+	+	0	0.12	0.17	5	-290.8	591.6	1.7
+	+	+	0.12	0.17	6	-290.8	593.6	3.7
0	0	0	0.00	0.04	3	-296.1	598.3	8.4
0	+	0	0.00	0.04	4	-296.0	600.0	10.1

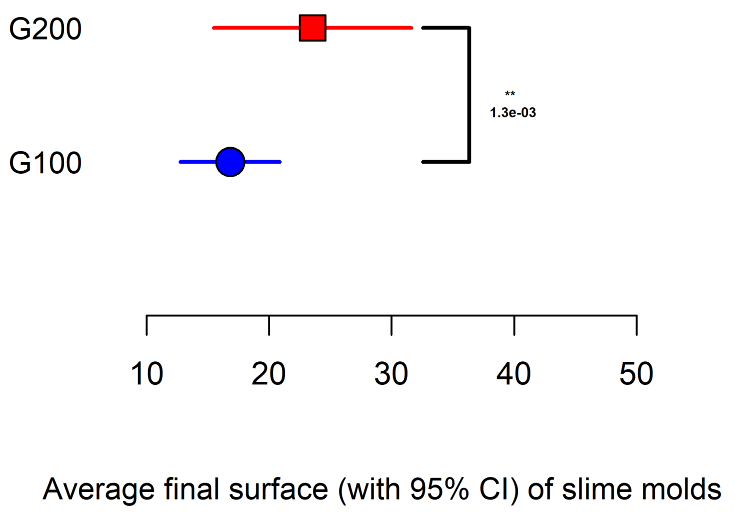

Figure 17 Average final surface (and 95% CI) of the selected model. The dendrogram represents a pairwise comparison using a Tukey test. The final surface of slime molds is higher when the furthest spot contains a highly nutritive patch than a slightly nutritive patch. The nearest patch has no effect on the final surface. Given the overlapping between the two confidence intervals, this result might be a false positive.

2. Mucus:

   a) Time elapsed until the first appearance

The surface threshold used to detect the first mucus production was 0.71 (pixels).

The cox model explaining the time elapsed until the first appearance by the nature of the treatment respected the proportional hazard ratio assumption.

Table 15: Model selection (using AIC) of cox models explaining the time elapsed until the first mucus appearance by the nature of the treatment in the spot experiments.

Food spot concentration	Obstacle	Food : Obstacle	R^2	df	logLik	AIC	delta
0	0	0	0.29	2	-260	523	0.0
+	0	0	0.29	3	-260	524	1.1
0	+	0	0.29	3	-260	525	1.8
+	+	0	0.30	4	-260	526	2.9
+	+	+	0.30	5	-259	528	4.6

There is no significant difference in the time elapsed until first mucus appearance among the different environments tested; the nature of the close patch and of the remote patch does not influence the occurrence time of mucus.

b) Growth rate during the linear growth at the beginning

The linear mixed model explaining the mucus growth showed no deviation from linear mixed models assumptions.

Table 16: Model selection (using AIC) of linear mixed models explaining the growth rate by the nature of the treatment in the spot experiments.

Food spot concentration	Obstacle	Food : Obstacle	R^2 for fixed effects	R^2 for all effects	df	logLik	AIC	delta
0	0	0	0.00	0.50	3	229.5	-453.1	0.000
+	0	0	0.01	0.51	4	230.2	-452.4	0.743
0	+	0	0.00	0.50	4	229.6	-451.1	1.991
+	+	+	0.02	0.53	6	231.5	-451.1	2.043
+	+	0	0.01	0.51	5	230.2	-450.4	2.734

There is no significant difference in the mucus growth rate among the different treatments. The mucus growth rate appears to be independent from the nature of the close patch and of that of the remote patch.

c) Final surface

The linear mixed model explaining the final surface by the different treatments showed a slight deviation of homoscedasticity and normality but linearity, independence and absence of outliers were respected.

Table 17: Model selection (using AIC) of linear mixed models explaining the final surface of mucus coverage by the nature of the treatment in the spot experiments.

Food spot concentration	Obstacle	Food : Obstacle	R^2 for fixed effects	R^2 for all effects	df	logLik	AIC	delta
+	0	0	0.09	0.27	4	-313.0	633.9	0.0
+	+	0	0.09	0.27	5	-312.9	635.8	1.9
+	+	+	0.09	0.27	6	-312.8	637.6	3.7
0	0	0	0.00	0.18	3	-317.4	640.9	7.0
0	+	0	0.00	0.18	4	-317.4	642.8	8.9

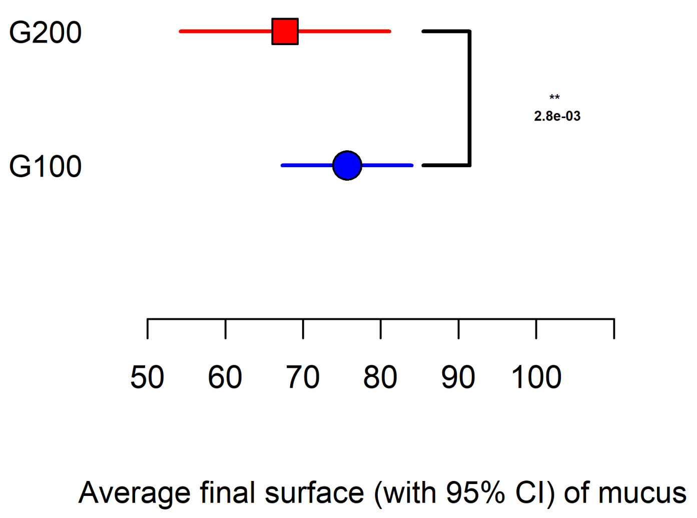

Figure 18: Average final surface (and 95% CI) from the selected model. The dendrogram represents a pairwise comparison using a Tukey test. The final surface

covered by mucus is lower when the furthest spot contains a highly nutritive patch than a slightly nutritive patch. The nearest patch has no significant effect on the final surface. Given the overlapping between the two confidence intervals, this result might be a false positive.

3. Total secondary growth

The linear mixed model explaining the total secondary growth showed no deviation from linear mixed models assumptions.

Table 18: Model selection (using AIC) of linear mixed models explaining the total secondary growth by the nature of the treatment in the spot experiments.

Food spot concentration	Obstacle	Food : Obstacle	R^2 for fixed effects	R^2 for all effects	df	logLik	AIC	delta
0	0	0	0.00	0.27	3	-523.1	1052.2	0.0
0	+	0	0.01	0.28	4	-522.8	1053.6	1.4
+	0	0	0.00	0.27	4	-523.1	1054.2	2.0
+	+	+	0.02	0.30	6	-521.8	1055.5	3.3
+	+	0	0.01	0.28	5	-522.8	1055.6	3.4

There is no significant difference in the total secondary growth among the different treatments. The total cumulated area covered by secondary growth does appear to be influenced by the nature of the close patch nor of that of the remote patch.

4. Total migration rate

The linear mixed model explaining the total migration rate displayed deviations from the linear mixed model assumptions because of 5 extreme values (whose mean was 45.4 mm/min; with these values the mean total migration rate was 129.9 mm/min and without, it was 135.6 mm/min). With and without these values, the statistical output remained the same. Since neither our set up nor our analysis were susceptible to be the cause of these values: we kept them for the following.

Table 19: Model selection (using AIC) of linear mixed models explaining variations in the total migration rate by the nature of the treatment in the spot experiments.

Food spot concentration	Obstacle	Food : Obstacle	R^2 for fixed effects	R^2 for all effects	df	logLik	AIC	delta
+	0	0	0.04	0.32	4	-367.6	743.1	0.0
+	+	0	0.04	0.32	5	-367.5	745.0	1.9
0	0	0	0.00	0.28	3	-369.6	745.3	2.2
+	+	+	0.04	0.32	6	-367.4	746.8	3.7
0	+	0	0.00	0.28	4	-369.6	747.2	4.1

The AIC difference between the null model and the model containing the food spot concentration factor is very low (2.2) but superior to 2.

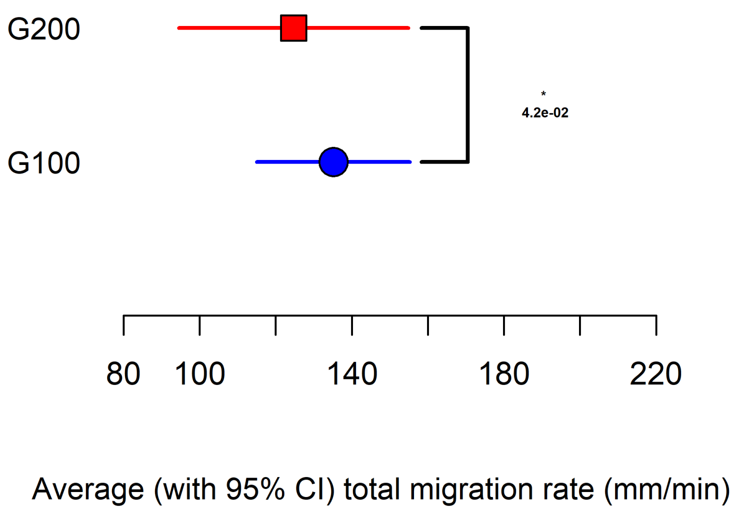

Figure 19: Average total migration rate (and 95% CI) from the selected model. The dendrogram represents a pairwise comparison using a Tukey test. While the obstacle had no effect, an increase in the food spot concentration involved a slight decrease in the total migration rate during the whole experiment. Given the overlapping between the two confidence intervals and the weak AIC delta, this result might be a false positive.

5. Tendency for secondary growth

The linear mixed model explaining the tendency for secondary growth displayed deviations from the linear mixed model assumptions because of 1 extreme value (69.54; with this value the mean tendency was 1.84 and without, it was 0.98). With and without this value, the statistical output remained the same. Since neither our set up nor our analysis were susceptible to be the cause of this value: we kept it for the following.

Table 19: Model selection (using AIC) of linear mixed models explaining variations in the tendency for secondary growth by the nature of the treatment in the spot experiments.

Food spot concentration	Obstacle	Food : Obstacle	R^2 for fixed effects	R^2 for all effects	df	logLik	AIC	delta
+	0	0	0.07	0.17	4	-319.7	647.4	0.0
+	+	0	0.08	0.17	5	-319.5	648.9	1.6
+	+	+	0.08	0.18	6	-319.2	650.3	3.0
0	0	0	0.00	0.09	3	-323.0	651.9	4.5
0	+	0	0.00	0.10	4	-322.8	653.5	6.1

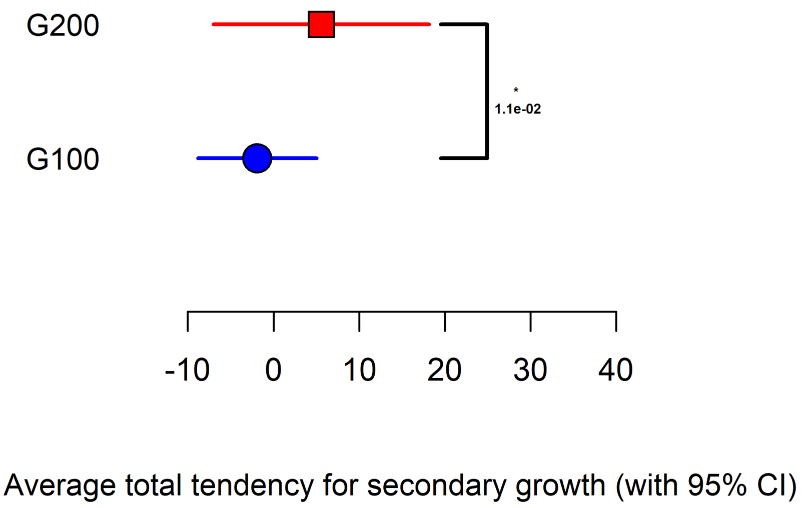

Figure 20: Average total tendency for secondary growth (and 95% CI) from the selected model. The dendrogram represents a pairwise comparison using a Tukey test. While the obstacle had no effect, an increase in the food spot concentration involved a slight increase in the total tendency for secondary. Given the overlapping between the two confidence intervals, this result might be a false positive.

6. Solidity decrease rate

When studying the decrease rate of solidity, our linear regression algorithm did produce an outlier value (leading to a positive slope). We removed that individual in order to not influence our analysis. By doing so, we also fixed deviations from the linear mixed model assumptions.

Table 20: Model selection (using AIC) of linear mixed models explaining variations in the decrease rate of solidity by the nature of the treatment in the spot experiments.

Food spot concentration	Obstacle	Food : Obstacle	R^2 for fixed effects	R^2 for all effects	df	logLik	AIC	delta
0	0	0	0.00	0.22	3	526.2	-1046.4	0
+	0	0	0.02	0.24	4	527.1	-1046.2	0.3
0	+	0	0.01	0.23	4	526.5	-1045.0	1.5
+	+	0	0.02	0.25	5	527.4	-1044.8	1.7

+	+	+	0.02	0.25	6	527.4	-1042.8	3.7

There is no significant difference in the solidity decrease rate among the different treatments. The solidity decrease rate does not seem to be influenced by the nature of the close patch nor of that of the remote patch.

7. New pseudopod number

When studying the new pseudopod number, the linear mixed model assumptions were respected.

Table 21: Model selection (using AIC) of linear mixed models explaining variations in the total number of new pseudopods that appeared during the spot experiment.

Food spot concentration	Obstacle	Food : Obstacle	R^2 for fixed effects	R^2 for all effects	df	logLik	AIC	delta
0	0	0	0.00	0.22	3	-363.2	732.3	0
+	0	0	0.02	0.24	4	-362.2	732.3	0
+	+	+	0.05	0.28	6	-360.3	732.6	0.3
0	+	0	0.00	0.22	4	-363.2	734.3	2.0
+	+	0	0.02	0.24	5	-362.2	734.3	2.0

8. Time to reach the food patch

The cox model explaining the time to reach a food patch by the treatments respected the proportional hazard ratio assumption.

Table 22: Model selection (using AIC) of cox models explaining variations in the time elapsed until slime mold reached a food patch by the nature of the treatment in the spot experiments.

Food spot concentration	Obstacle	Food : Obstacle	R^2	df	logLik	AIC	delta
+	+	0	0.56	4	-241	489	0.0
+	+	+	0.56	5	-241	491	1.7
+	0	0	0.53	3	-244	492	3.0
0	+	0	0.50	3	-246	497	7.9

0	0	0	0.47	2	-249	500	11.1

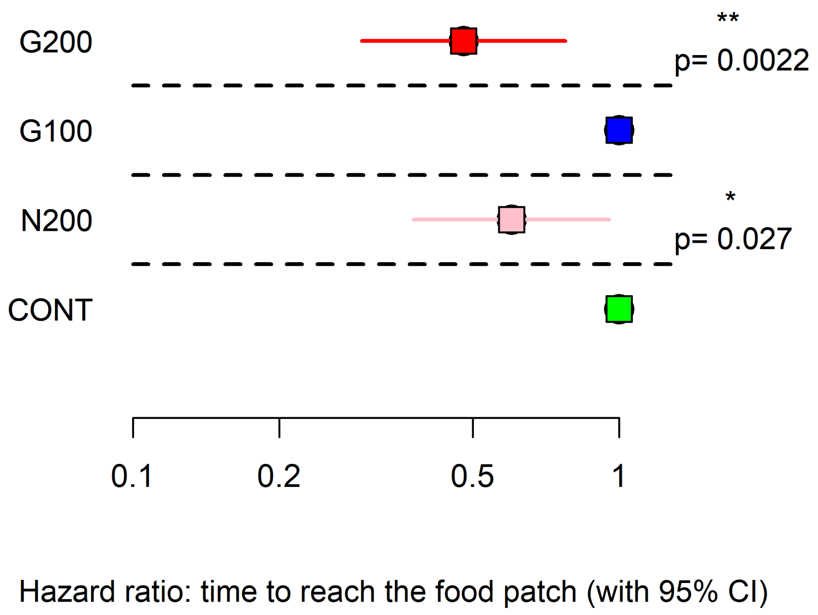

Figure 21: Hazard ratio (and 95% CI) of the fixed effects of the selected model. The lower the hazard ratio, the lower the risk –at any time- that the slime mold reaches the food patch, i.e. the lower the hazard ratio, the more time needed by slime molds to reach the food patch. The two tested factors have a significant impact on the time to reach the food patch: When the nearest food patch contains 100 mM NaCl instead of only agar, and when the furthest food patch contains 200 mM glucose instead of 100 mM glucose, slime molds take more time to reach the food patch.

IV. References

1. Team, R. C. & others 2013 R: A language and environment for statistical computing.

2. Therneau, T. M. & Therneau, M. T. M. 2015 Package “coxme.”*R package version* **2**.

3. Therneau, T. M. & Lumley, T. 2014 Package “survival.”*Survival analysis Published on CRAN*

4. Bates, D., Sarkar, D., Bates, M. D. & Matrix, L. 2007 The lme4 package. *R package version* **2**, 74.

5. Kuznetsova, A., Brockhoff, P. B., Christensen, R. H. B. & others 2015 Package “lmertest.”*R package version* **2**.

6. Barton, K. & Barton, M. K. 2015 Package “MuMIn.”*Version* **1**, 18.

7. Lenth, R. 2018 Emmeans: Estimated marginal means, aka least-squares means. *R package version* **1**.

S2 Video. Time-lapse of one experiment replicate, original images.

Video showing the evolution of the slime mold cell over the 35 hours of the experiments, original acquired photos.

S3 Video. Time-lapse of one experiment replicate, trinarized images.

Time lapse of the results of the image segmentation for the same experiment shown in S2 Video.

S4 Appendix: Statistic analysis script description

This script summarizes the procedure that we used to analyze the morphogenesis and dynamics of slime molds in various environments. This document does not provide the exhaustive list of analyses that we did, but rather an instanciation of our approach. Besides, this script contains a function (called “find.curves.features”), which can be used to do a time series analysis, and allows finding more information than presented in the paper.

This code requires the following R packages: reshape2, ggplot2, lme4, lmerTest, survival, coxme, MuMIn, emmeans

First, set the working directory and load required packages

```
setwd("G:/Directory/scripts_n_tables/GT/final_tables/")
```

Script Table of Contents

1. Temporal dynamics

   1.1 Threshold for first movement/occurrence detection (a homogeneous experiment instance)

   1.2 Growth features extraction algorithm: first movement, best slope, time and surface at plateau, final value

   1.3 Total secondary growth (a homogeneous experiment instance)

   1.4 Trend towards secondary growth (a homogeneous experiment instance)

   1.5 New pseudopod number calculation (a homogeneous experiment instance)

   1.6 Time to reach the food patch (spot experiment)

2. Statistical procedure

   2.1 A linear mixed model instance: the trend towards secondary growth in the homogeneous experiment

   2.2 A cox mixed model instance: the time to reach a nutritive patch in the spot experiment

1. Temporal dynamics

1.1 Threshold for first movement/occurrence detection (a homogeneous experiment instance)

Load the table containing the dynamics of the homogeneous experiment

```
homogen_dyn_raw = read.csv("homogeneous_dynamics_rawdata.csv")
```

Select one temporal variable (here, slime mold surface against time) and organize it with one replicate per column

```
slime_mold_dyn = homogen_dyn_raw[,"SlimeMold"]
slime_mold_dyn_by_replicate = matrix(slime_mold_dyn,ncol=20,nrow=462)
```

Calculate for each replicate, 1% of the difference between the maximal and minimal surface

```
one_percent_max_surface_increase_per_replicate = 0.01*abs(apply(slime_mold_dyn_by_replic
ate,2,max)-apply(slime_mold_dyn_by_replicate,2,min))
```

By averaging between replicates, get the surface threshold allowing the detection of the slime mold first movement and the mucus first occurrence

```
surface_threshold = mean(one_percent_max_surface_increase_per_replicate)
```

This threshold can be used in the next section in order to determine the time elapsed until the first movement/occurrence.

1.2 Growth features extraction algorithm: first movement, best slope, time and surface at plateau, final value

Set the time between each slime mold picture during the experiment

```
time_step = 5
```

Select one individual to analyze

```
individual_to_analyze = slime_mold_dyn_by_replicate[,17]
```

Load the “find curve features” function

```
find.curves.features <- function(y, time_step, surface_threshold, first_frame=1, noisy=F){

##### a) Determine the growth period limits to improve speed (i.e. look for only relevent regres
sions)

# Define if the shape of the curve is decreasing or increasing
  if(surface_threshold<0){shape="decreasing"}else{shape="increasing"}

# Define the total duration of the vector y
  n_frame <- length(y);nn_size = c(n_frame,n_frame)

# The variable must be numeric for regression
  y <- as.numeric(y)
```

```
# What is the maximal difference in y
  maximal_scope = abs(max(y) - min(y))

# We consider the begining of a growth when it has reached a given "surface_threshold"
  if (shape=="increasing"){
    first_mov = which((y > y[2]+surface_threshold))[1]
  } else {
    first_mov = which((y < y[2]+surface_threshold))[1]
  }

# Calculate clustered means to detect slope shifts:
# We gather data in clusters and look for the sign evolution of the mean of each cluster
  cluster_length <- 5 ; cluster_nb <- floor(n_frame/(cluster_length))
  clust_mean_diffs <- diff(apply(matrix(y,ncol=cluster_nb,nrow=cluster_length),2,mean))
  if (shape=="increasing"){
    slope_shifts <- which(clust_mean_diffs<mean(diff(y)))*cluster_length
  }else{
    slope_shifts <- which(clust_mean_diffs>mean(diff(y)))*cluster_length
  }

# Look for the moment when the slope did shift and y reached 90% of the maximal scope to deter
mine the plateau
  if(any(slope_shifts %in% which(y>y[2]+0.9*maximal_scope))){
    if (shape=="increasing"){
      plateau_time <- slope_shifts[slope_shifts %in% which(y>(y[2]+0.9*maximal_scope))][1]
    }else{
      plateau_time <- slope_shifts[slope_shifts %in% which(y<(y[2]-0.9*maximal_scope))][1]
    }
    max_end <- plateau_time
  }else{
    plateau_time <- "censored"
    max_end <- n_frame
  }

# If the first frame is different from the last,
  if (y[1]!=y[n_frame]){

# Set the maximal frame to compute linear and exponential regressions
    if (shape=="increasing"){
      max_start <- which(y > (y[2] + 0.1*maximal_scope))[1]
      max_exp_start <- which(y > (y[2] + 0.01*maximal_scope))[1]
    }else{
      max_start <- which(y < (y[2] - 0.1*maximal_scope))[1]
      max_exp_start <- which(y < (y[2] - 0.01*maximal_scope))[1]
    }

# Creates 0-containing matrices whose dimensions correspond to the first (rows) and the last (col
```

```
umns) frame of regressions
   exp_rsquared = array(0,dim=nn_size) # Will contain exponential regression R²
   lin_rsquared = array(0,dim=nn_size) # Will contain linear regression R²

# replace the null y values by the smalest non-null y value to avoid singular fits (impossibility of lo
g(0))
   y[y==0] <- y[y[order(y)]!=0][1]

# try every possibilities of time combination to begin and end each regression
   for(frame_i in 1:max_start) {
    for(frame_j in max_start:max_end) {

# For every regression, only calculate those contanining an increase/decrease larger than 50% of
the maximal scope
      if(abs(y[frame_j]-y[frame_i])>0.5*maximal_scope) {

# Build the time axis
        Time <- seq((frame_i-1)*time_step,(frame_j-1)*time_step,time_step)

### Storage of exponential regression R²
        if(frame_i<max_exp_start){
          if(shape=="decreasing"){
            y_neg_exp <- -y + 2*max(y)
            exp_reg <- lm(log(y_neg_exp[c(frame_i:frame_j)]) ~ Time)
          }else{
            exp_reg <- lm(log(y[c(frame_i:frame_j)]) ~ Time)
          }
          exp_rsquared[frame_i,frame_j] <- summary(exp_reg)$r.squared
        }

### Storage of linear regression R²
        lin_reg <- lm(y[c(frame_i:frame_j)] ~ Time)
        lin_rsquared[frame_i,frame_j] <- summary(lin_reg)$r.squared
      }
    }
   }

# Replace NA with 0
   exp_rsquared[is.na(exp_rsquared)] <- 0 ; lin_rsquared[is.na(lin_rsquared)] <- 0

# Selected exponential regression than best describe the data
   exp_best_length <- arrayInd(which.max(exp_rsquared),nn_size)
   exp_Time <- seq((exp_best_length[1]-1)*time_step,(exp_best_length[2]-1)*time_step,time_s
tep)
   if(shape=="decreasing"){
    y_neg_exp <- -y + 2*max(y)
    best_exp_modl <- lm(log(y_neg_exp[c(exp_best_length[1]:exp_best_length[2])])) ~ exp_Time
)
```

```
   }else{
     best_exp_modl <- lm(log(y[c(exp_best_length[1]:exp_best_length[2])]) ~ exp_Time)
   }

# Selected linear regression than best describe the data
   lin_best_length <- arrayInd(which.max(lin_rsquared),nn_size)
   lin_Time <- seq((lin_best_length[1]-1)*time_step,(lin_best_length[2]-1)*time_step,time_step)
   best_lin_modl <- lm(y[c(lin_best_length[1]:lin_best_length[2])] ~ lin_Time)

# If the plateau detection algorithm failed to detect one: quote it as censored
   if (plateau_time=="censored"){plateau_value<-"censored"}

# Also store the surface at which the plateau has been reached
   if (plateau_time!="censored"){plateau_value <- y[plateau_time] ; plateau_time <- time_step*
plateau_time}

# Gather every feature from the analysis in one vector
   output <- c(time_step*first_mov, best_exp_modl$coefficients[1:2], time_step*exp_best_leng
th[1:2],
            best_lin_modl$coefficients[1:2], time_step*lin_best_length[1:2],
            plateau_time, plateau_value, max(exp_rsquared),max(lin_rsquared),y[length(y)])
  }else{output <- c(ifelse(is.na(first_mov),0,first_mov),c(rep(0,14-1)))}

# Display the dot cloud
  par(mar=c(4, 4, 1,1))
  Time <- seq(0,(n_frame-1)*time_step,time_step)
  plot(Time,y, ylab="Surface (pixels)",xlab="Time (min)")

# Add features to the dot could
# 1. The first movement red line
  abline(v=output[1], col="red",lwd=2)

# 2. The exponential purple curve
  lines(Time, exp(output[2] + (output[3]*Time)),lwd=2, col = "purple", xlab = "Time (s)", ylab = "C
ounts")

# 3. The exponential dotted limits
  abline(v=output[4], col="purple",lty=6,lwd=2)
  abline(v=output[5], col="purple",lty=6,lwd=2)

# 4. The linear orange curve
  lines(Time, output[6]+(output[7]*Time),lwd=2, col = "orange", xlab = "Time (s)", ylab = "Counts
")

# 5. The linear dotted limits
  abline(v=output[8], col="orange",lty=3,lwd=2)
  abline(v=output[9], col="orange",lty=3,lwd=2)
```

```
# 6. The plateau coordinates (if reached)
  if(plateau_time!="censored")
  {
   abline(v=output[10], col="dodgerblue",lwd=2)
   abline(h=output[11], col="dodgerblue",lwd=2)
  }

# Legend:
  legend("bottomright", paste(c("First movement","Exponential R²","Linear R²", "Plateau"),  as.c
haracter(c(paste(output[1]," min",sep=" "),format(as.numeric(output[c(12:13)]),digits=2),paste(
output[10]," min",sep=" "))),sep=": "), text.col=c("red","purple","orange","dodgerblue"))

# Give names to the output values
   names(output)[]<- c("first_movement", "exp_intercept","exp_growth_rate","exp_start","exp_
end","linear_intercept","linear_slope","lin_start","lin_end","growth_rupture_time","growth_ru
pture_surface","exp_rsquared","linear_rsquared","final_value")

  return(output)
}
```

Use the function on the surface evolution of one slime mold:

```
find.curves.features(individual_to_analyze, time_step, surface_threshold)
```

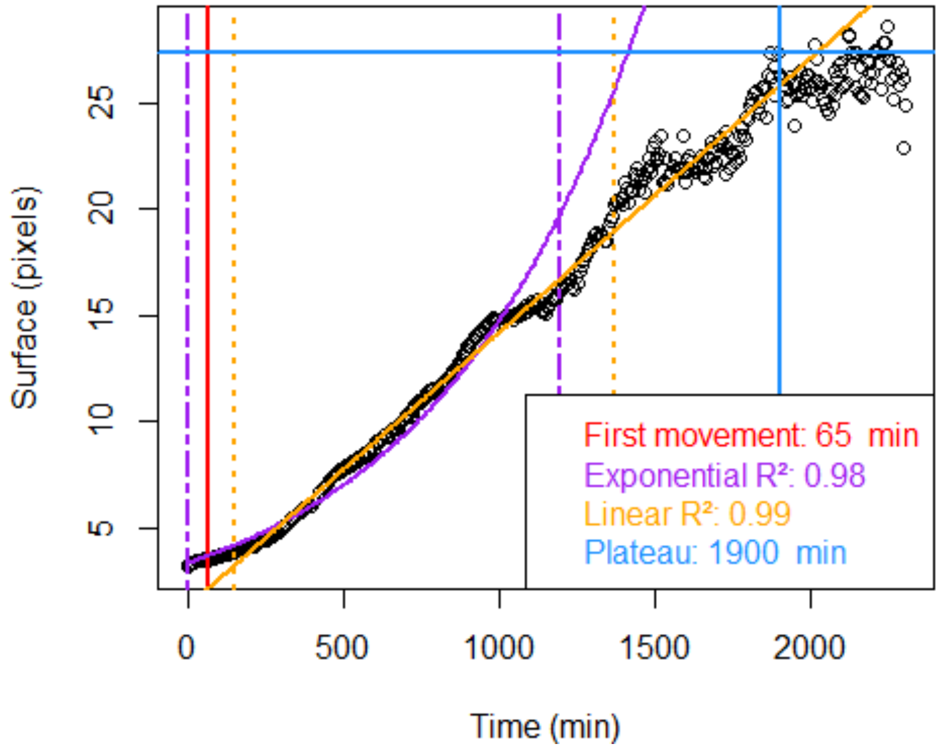


```
##        first_movement        exp_intercept       exp_growth_rate
##         6.500000e+01         1.199274e+00         1.491829e-03
```

```
##            exp_start              exp_end       linear_intercept
##         5.000000e+00         1.195000e+03          1.260563e+00
##         linear_slope            lin_start              lin_end
##         1.293231e-02         1.550000e+02          1.370000e+03
##   growth_rupture_time growth_rupture_surface          exp_rsquared
##         1.900000e+03         2.738323e+01          9.763199e-01
##       linear_rsquared          final_value
##         9.925270e-01         2.488478e+01
```

1.3 Total secondary growth (a homogeneous experiment instance)

Load the table containing the dynamics of the homogeneous experiment

```
Secondary_growth_dyn = homogen_dyn_raw[,c("Date","Treatment","Time","SecondaryGrowth_cum")]
```

For each individual, extract the last value of the cumulated secondary growth time dynamic

```
Secondary_growth_total = secondary_growth_dyn[secondary_growth_dyn$Time==2310,]
```

1.4 Trend towards secondary growth calculation (a homogeneous experiment instance)

Load the table containing the study of the behavioral trends of the homogeneous experiment

```
Homogen_tendencies_raw = read.csv("homogeneous_tendencies_rawdata.csv")
```

Use the dcast function from the package reshape2 to build a table containing the time evolution of the secondary growth proportion:

```
Realized_secondary_growth <- dcast(homogen_tendencies_raw,Treatment+Date+Replicate~Time,value.var="Secondary_Growth__percent")
```

And the time evolution of the expected secondary growth, i.e. the proportion of pixels, near the slime mold, that are already covered with mucus.

```
Expected_secondary_growth <- dcast(homogen_tendencies_raw,Treatment+Date+Replicate~Time,value.var="Mucus__percent")
```

Calculate, for each slime mold, the trend towards secondary growth by making the total sum of the differences between the actual and the expected secondary growth

```
Tendency <-realized_secondary_growth[,1:3]
for(i in 1:nrow(tendency)){
  tendency[i,4] <- sum(as.numeric(realized_secondary_growth[i,4:ncol(realized_secondary_growth)])
                  -as.numeric(expected_secondary_growth[i,4:ncol(realized_secondary_growth)]))
}
names(tendency)[4] = "tendency_for_secondary_g"
```

1.5 New pseudopod number calculation (a homogeneous experiment instance)

Use the dcast function from the package reshape2 to build a table containing the time evolution of the number of pseudopods:

```
pseudopod_number_dynamics <- dcast(homogen_dyn_raw,Treatment+Replicate+Date~Time,va
lue.var="NumClusters")
```

Substract the number of pseudopods at time t from the number of pseudopods at time t+1:

```
pseu_dim = dim(pseudopod_number_dynamics)
pseudopod_increasing_number =  array(dim=c(pseu_dim-c(0,4)))
pseudopod_increasing_number[,c(1:(pseu_dim[2]-4))] = mapply("-",pseudopod_number_dyna
mics[,c((4+1):(pseu_dim[2]))],pseudopod_number_dynamics[,c(4:(pseu_dim[2]-1))])
```

Keep only the increasing number of pseudopods i.e. the positive values

```
pseudopod_increasing_number[pseudopod_increasing_number<0] = 0
```

Calculate the total sum of the increasing number of pseudopods

```
new_pseudopod_number = cbind(pseudopod_number_dynamics[,c(1:3)],apply(pseudopod_inc
reasing_number,1,sum))
names(new_pseudopod_number)[4] = "new_pseudopods"
```

1.6 Time to reach the food patch calculation (spot experiment)

Load the table containing the dynamics of the homogeneous experiment

```
spot_dyn_rawdata = read.csv("spot_dynamics_rawdata.csv")
```

Use the dcast function from the package reshape2 to build a table containing the time evolution of the distance from a food patch

```
food_patch_distance = dcast(spot_dyn_rawdata,near_spot+far_spot+Date+Replicate~Time,valu
e.var="DistToFood")
```

For each individual, detect the first time frame when the distance from the food patch reaches 0

```
reach_food = numeric(nrow(food_patch_distance))
for(i in 1:nrow(food_patch_distance)){
  reach_food[i] <- which(food_patch_distance[i,c(5:ncol(food_patch_distance))]==0)[1]
}
time_to_reach_food = cbind(food_patch_distance[,c(1:5)],reach_food)
names(time_to_reach_food)[6] = "reach_food"
```

2. Statistical procedure

2.1 A linear mixed model instance: the trend towards secondary growth in the homogeneous experiment

Use the lmer function in the lme4 package to build the statistical model explaining the trend towards secondary growth with the treatment as a fixed effect and the Date as a random effect.

```
tendency_model <- lmer(tendency_for_secondary_g ~ Treatment + (1|Date),REML=F, data = ten
dency)
```

Verify the model adequacy

1. By looking at the correlations in the summary: none of them are near to one

```
summary(tendency_model)

## Linear mixed model fit by maximum likelihood . t-tests use
##   Satterthwaite's method [lmerModLmerTest]
## Formula: tendency_for_secondary_g ~ Treatment + (1 | Date)
##    Data: tendency
##
##      AIC      BIC   logLik deviance df.resid
##    684.0    698.3   -336.0    672.0       74
##
## Scaled residuals:
##     Min      1Q  Median      3Q     Max
## -2.2747 -0.5415 -0.0808  0.5995  3.9833
##
## Random effects:
##  Groups   Name        Variance Std.Dev.
##  Date     (Intercept)  61.72    7.856
##  Residual             237.81   15.421
## Number of obs: 80, groups:  Date, 4
##
## Fixed effects:
##              Estimate Std. Error      df t value Pr(>|t|)
## (Intercept)     3.7770     5.2318  8.6431   0.722    0.489
## TreatmentG100  -0.3661     4.8766 75.9798  -0.075    0.940
## TreatmentG200  28.0788     4.8766 75.9798   5.758 1.71e-07 ***
## TreatmentN100  54.2314     4.8766 75.9798  11.121  < 2e-16 ***
## ---
## Signif. codes:  0 '***' 0.001 '**' 0.01 '*' 0.05 '.' 0.1 ' ' 1
##
## Correlation of Fixed Effects:
##             (Intr) TrG100 TrG200
## TretmntG100 -0.466
## TretmntG200 -0.466  0.500
## TretmntN100 -0.466  0.500  0.500
```

We also see here that the slightly nutritive environment is not different from the neutral environment. However, in the adverse and highly nutritive environments, slime molds have a higher tendency for secondary growth

2. By verifying linearity and homoscedasticity

```
plot(tendency_model)
```

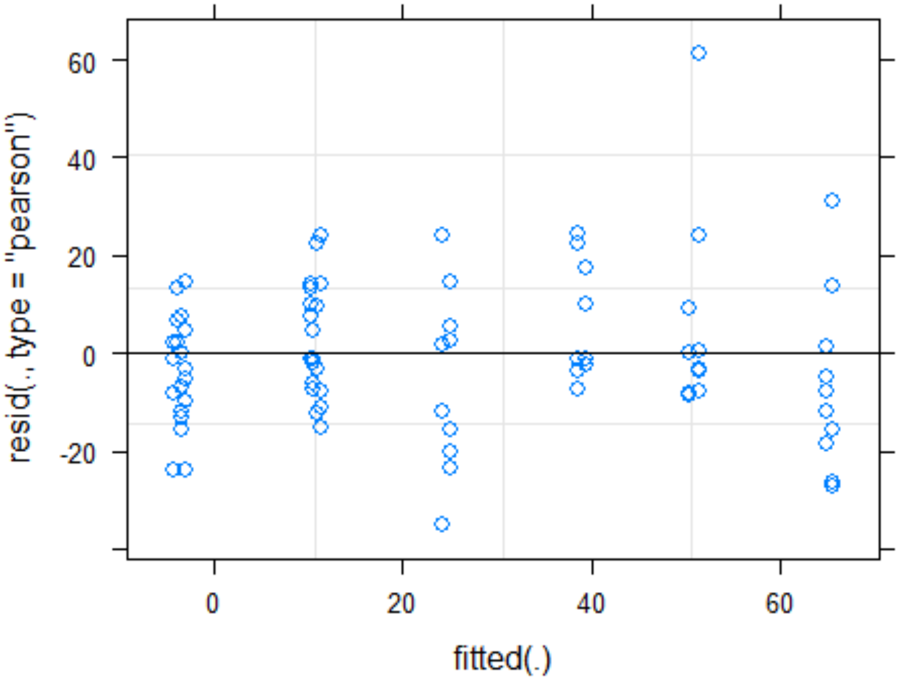

3. By verifying normality

```
qqnorm(residuals(tendency_model))
```

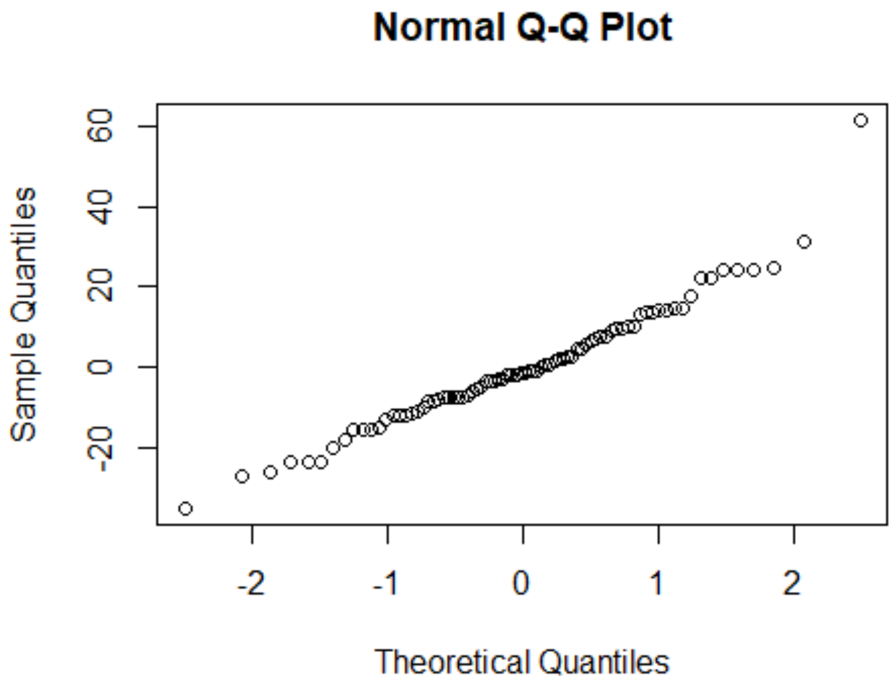

4. By looking for eventual outliers

```
ggplot(data.frame(lev=hatvalues(tendency_model),pearson=residuals(tendency_model,type="pearson")),
        aes(x=lev,y=pearson)) +  geom_point() +  theme_bw()
```

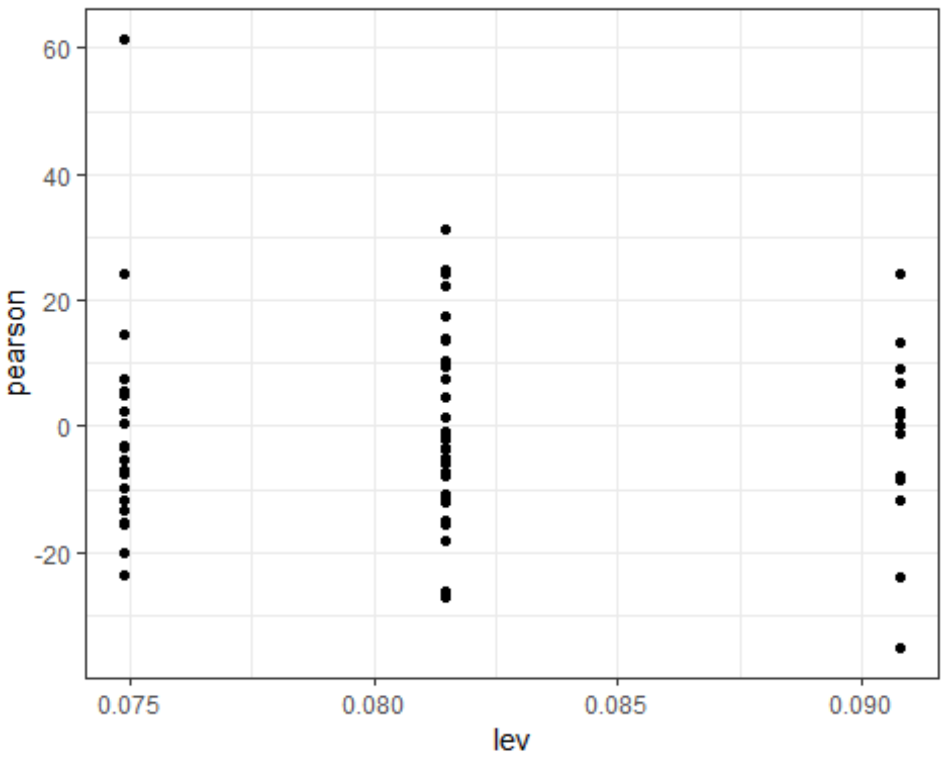

Since assumptions are not strongly violated, proceed to model comparison
Model comparisons”
Using the MuMIn package, compare every potential nested model in the full model using the AIC method

```
options(na.action = "na.fail")
dredge(tendency_model, rank = "AIC", extra = c("r.squaredGLMM"))

## Fixed term is "(Intercept)"

## Global model call: lmer(formula = tendency_for_secondary_g ~ Treatment + (1 | Date),
##     data = tendency, REML = F)
## ---
## Model selection table
##   (Intrc) Trtmn r.squaredGLMM1 r.squaredGLMM2 df   logLik AIC delta weight
## 2   3.777     +        0.6342       0.70950  6 -336.006 684  0.00      1
## 1  24.310              0.0000       0.04171  3 -381.016 768 84.02      0
## Models ranked by AIC(x)
## Random terms (all models):
## '1 | Date'

options(na.action = "na.omit")
```

The selected model does contain the Treatment factor.
Pairwise comparison of the levels of the Treatment factor

```
paiwise_tendency = emmeans(tendency_model, "Treatment", adjust = "tukey")
summary(pairs(paiwise_tendency))
```

```
##  contrast    estimate   SE df t.ratio p.value
##  CONT - G100    0.366 4.88 76   0.075 0.9998
##  CONT - G200  -28.079 4.88 76  -5.758 <.0001
##  CONT - N100  -54.231 4.88 76 -11.121 <.0001
##  G100 - G200  -28.445 4.88 76  -5.833 <.0001
##  G100 - N100  -54.597 4.88 76 -11.196 <.0001
##  G200 - N100  -26.153 4.88 76  -5.363 <.0001
##
## P value adjustment: tukey method for comparing a family of 4 estimates
```

Detection of another significant difference: slime molds on an adverse environment have a higher bias towards secondary growth than on any other environment (even on a highly nutritive environment).

2.2 A cox mixed model instance: the time to reach a nutritive patch in the spot experiment

Use the coxme function in the coxme package to build the statistical model explaining the time to reach a nutritive patch with the nature of the obstacle (near_patch), the food patch concentration (far_patch) as a fixed effects and the Date as a random effect.

```
reach_food_model = coxme(Surv(reach_food) ~ near_spot * far_spot + (1|Date), data = time_to
_reach_food)
```

Verify the model adequacy

By verifying the proportional hazard assumption on the equivalent cox model

```
reach_food_model2 <- coxph(Surv(reach_food) ~ near_spot * far_spot + Date, data = time_to_r
each_food)
test_ph<-cox.zph(reach_food_model2)
plot(test_ph)
```

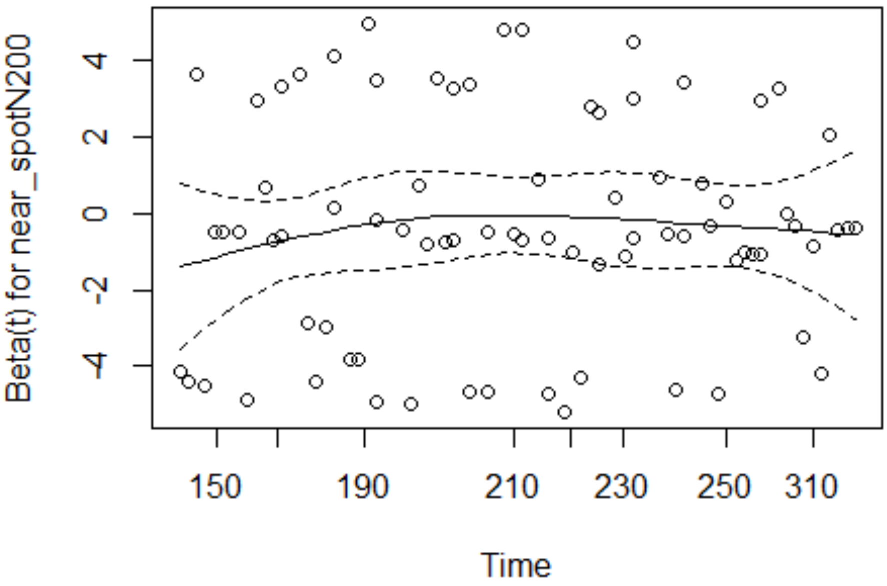
Beta(t) for near_spotN200
4
2
0
-2
-4
150
190
210
230
250
310
Time

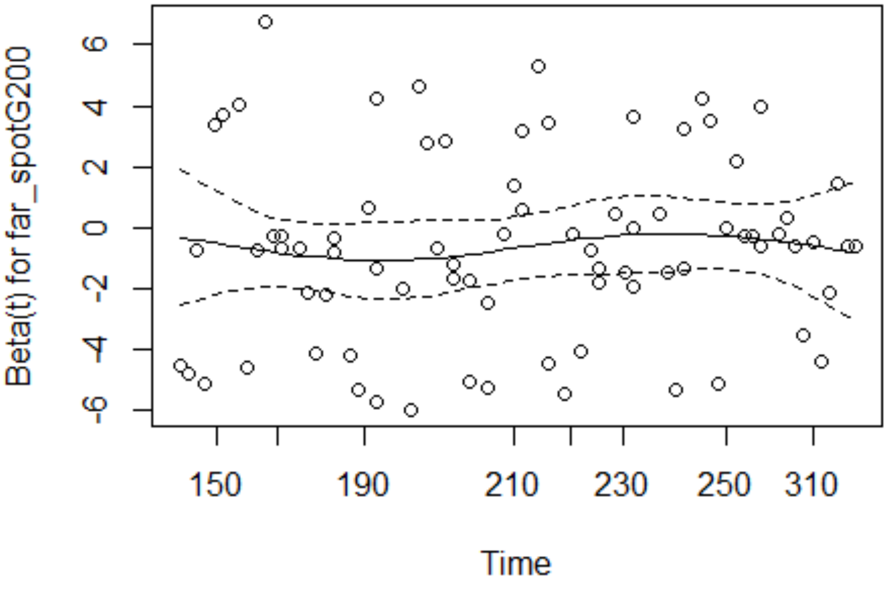
Beta(t) for far_spotG200
6
4
2
0
-2
-4
-6
150
190
210
230
250
310
Time

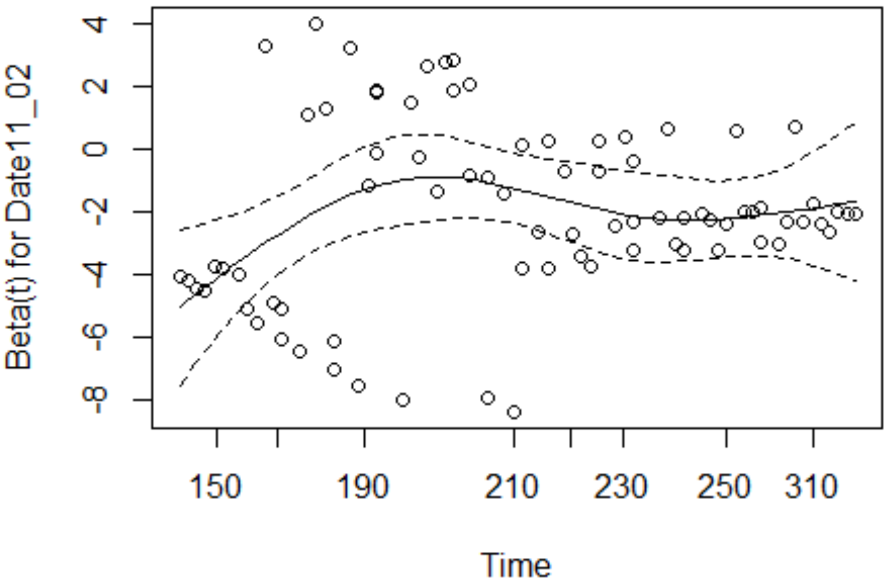

Beta(t) for Date11_02
4
2
0
-2
-4
-6
-8
150
190
210
230
250
310
Time

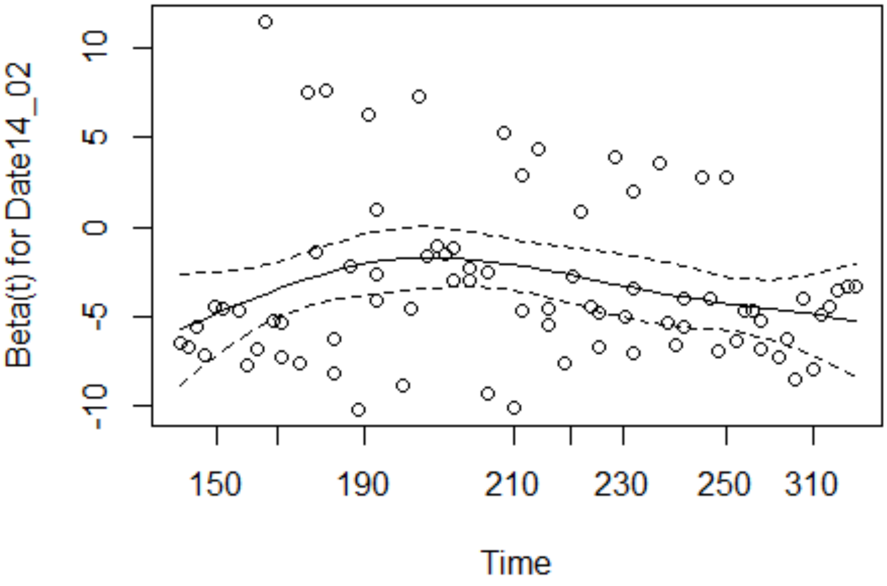

Beta(t) for Date14_02
10
5
0
-5
-10
150
190
210
230
250
310
Time

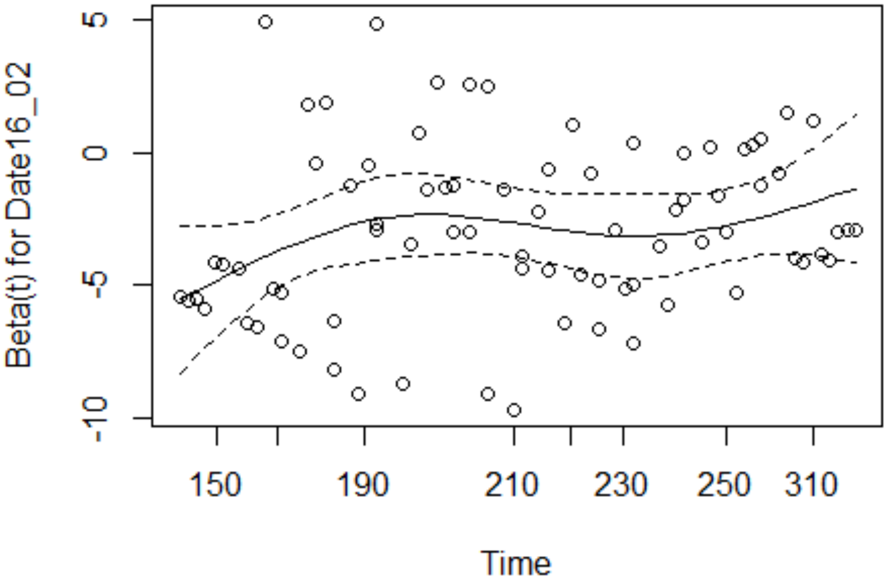

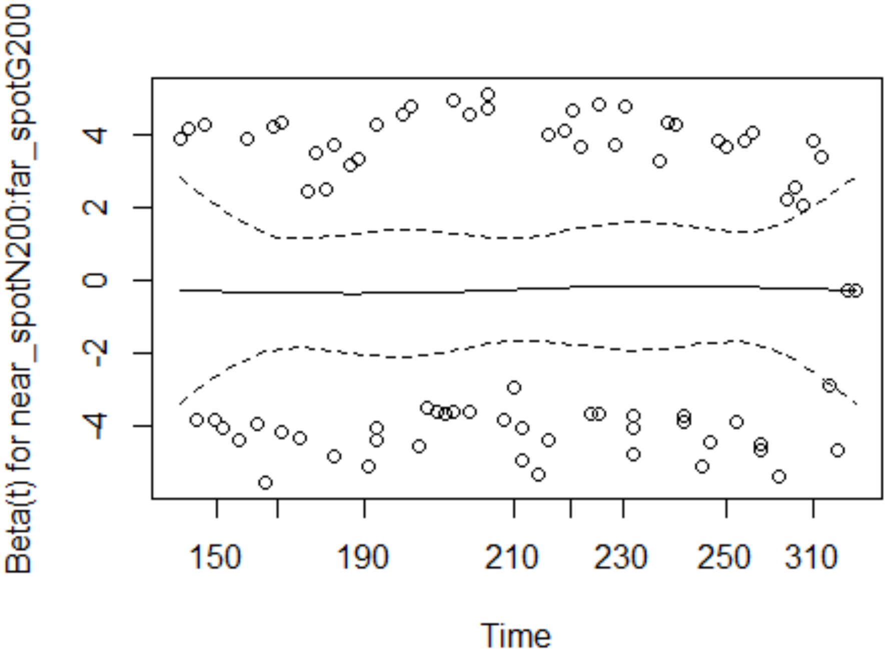

Since the proportional hazard assumption is not strongly violated, proceed to model comparison
Model comparisons
Using the MuMIn package, compare every potential nested model in the full model using the AIC method

```
options(na.action = "na.fail")
dredge(reach_food_model, rank = "AIC", extra = "R^2")

## Global model call: coxme(formula = Surv(reach_food) ~ near_spot * far_spot + (1 |
##     Date), data = time_to_reach_food)
## ---
## Model selection table
##   far_spt ner_spt far_spt:ner_spt    R^2 df   logLik   AIC delta weight
## 4       +       +                 0.5580  4 -241.019 489.0  0.00  0.593
## 8       +       +               + 0.5598  5 -240.850 490.6  1.66  0.258
## 2       +                         0.5296  3 -243.503 491.9  2.96  0.135
## 3               +                 0.4998  3 -245.965 496.9  7.87  0.012
## 1                                 0.4657  2 -248.598 500.1 11.13  0.002
## Models ranked by AIC(x)
## Random terms (all models):
## '(1 | Date)'

options(na.action = "na.omit")
```

The selected model does contain the nature of the obstacle (neutral vs adverse) and the concentration of the food patch (slightly vs highly nutritive).

```
reach_food_model3 = coxme(Surv(reach_food) ~ near_spot + far_spot + (1|Date), data = time_t
o_reach_food)
summary(reach_food_model3)

## Cox mixed-effects model fit by maximum likelihood
##   Data: time_to_reach_food
##   events, n = 80, 80
##   Iterations= 8 38
##                     NULL Integrated    Fitted
## Log-likelihood -273.6731  -246.2415 -239.5891
##
##                   Chisq  df          p   AIC   BIC
## Integrated loglik 54.86 3.0 7.3429e-12 48.86 41.72
##  Penalized loglik 68.17 4.9 2.1350e-13 58.37 46.70
##
## Model:  Surv(reach_food) ~ near_spot + far_spot + (1 | Date)
## Fixed coefficients
##                    coef exp(coef)  se(coef)     z      p
## near_spotN200 -0.510221 0.6003629 0.2313929 -2.20 0.0270
## far_spotG200  -0.736645 0.4787173 0.2410792 -3.06 0.0022
##
## Random effects
##  Group Variable  Std Dev  Variance
##  Date  Intercept 1.469914 2.160648
```

The presence of a salt obstacle and/or the increase of glucose concentration in the food patch lead(s) to a decrease -at any time- of the probability to reach the food patch, i.e. reaching the food patch takes more time when there is an adverse obstacle and/or when the nutritive patch concentration increases.
